# Clinical Characteristics and Survival Outcomes of a Clinically Defined Treatment-Emergent Neuroendocrine/Aggressive-Variant Prostate Cancer Phenotype: A Retrospective Study

**DOI:** 10.3390/medicina62091702

**Published:** 2026-09-05

**Authors:** Hakan Taban, Sercan Aksoy, Deniz Can Güven, Burak Yasin Aktaş, Feride Yılmaz, Ferit Aslan, Mustafa Erman

**Affiliations:** 1 Medical Oncology Clinic, Medical Park Ankara Hospital, 06680 Ankara, Turkey; feritferhat21@gmail.com; 2 Department of Medical Oncology, Hacettepe University Cancer Institute, 06230 Ankara, Turkey; saksoy07@yahoo.com (S.A.); denizcguven@hotmail.com (D.C.G.); byaktas@hotmail.com (B.Y.A.); 3 Medical Oncology Clinic, Samsun Education and Research Hospital, 55090 Samsun, Turkey; doktorferide@gmail.com; 4 Department of Preventive Oncology, Hacettepe University Cancer Institute, 06230 Ankara, Turkey; ermanm1968@gmail.com

**Keywords:** metastatic castration-resistant prostate cancer, neuroendocrine prostate cancer, aggressive-variant prostate cancer, platinum-based chemotherapy, prognostic factors

## Abstract

*Background and Objectives*: Treatment-emergent neuroendocrine prostate cancer (t-NEPC) is an aggressive resistance phenotype arising during metastatic castration-resistant prostate cancer (mCRPC). Because metastatic biopsy is not routinely feasible in advanced disease, real-world data on clinically defined t-NEPC/aggressive-variant prostate cancer (AVPC)-like disease remain limited. We aimed to characterize the clinical features, treatment patterns, survival outcomes, and prognostic factors of this clinically defined phenotype. *Materials and Methods*: We retrospectively reviewed 354 patients with prostate cancer treated at our institution between 2010 and 2020. Seventy-four patients with mCRPC who were clinically identified as having a t-NEPC/AVPC-like phenotype and had a treatment plan for platinum- and/or etoposide-based neuroendocrine-directed systemic therapy were included. Overall survival (OS) and radiographic progression-free survival (rPFS) were estimated using the Kaplan–Meier method, and prognostic factors were evaluated using Cox regression analyses. *Results*: At clinical identification of the phenotype, the median age was 66.4 years, and visceral metastases were present in 67.6% of patients, most commonly in the liver (55.4%). Median intervals from prostate cancer diagnosis and CRPC onset to clinical identification of the phenotype were 47.5 and 22.7 months, respectively. Neuroendocrine-directed therapy was initiated in 72 patients; platinum–etoposide was the most common first-line regimen (*n* = 41, 55.4%). Median OS was 4.4 months (95% confidence interval [CI], 2.9–5.8), and median rPFS was 3.5 months (95% CI, 2.7–4.2). In an exploratory, unadjusted analysis, median OS did not differ significantly between doublet chemotherapy and monotherapy (7.0 vs. 3.5 months; *p* = 0.167). Gleason score ≥9, hemoglobin <12 g/dL, and albumin <3.5 g/dL were independently associated with inferior OS. *Conclusions*: The clinically defined t-NEPC/AVPC-like phenotype was associated with an aggressive clinical course and poor survival. Earlier recognition, improved biomarker-based diagnostic strategies, and more effective therapeutic approaches are needed.

## 1. Introduction

Prostate cancer is the second most frequently diagnosed malignancy and the fifth leading cause of cancer-related death among men worldwide, accounting for approximately 1.5 million new cases and nearly 400,000 deaths in 2022 [1]. Although most patients are diagnosed with localized disease, a subset presents with or subsequently develops metastatic disease, which remains incurable and is associated with substantial morbidity and mortality [2]. Patients with metastatic prostate cancer initially respond to androgen deprivation therapy (ADT), often combined with docetaxel or an androgen receptor pathway inhibitor (ARPI); however, many eventually progress to metastatic castration-resistant prostate cancer (mCRPC) [2,3]. While resistance in most patients continues to depend on persistent or reactivated androgen receptor (AR) signaling, a clinically important subset develops AR-independent progression through lineage plasticity and neuroendocrine differentiation [4,5,6]. This treatment-resistant phenotype is commonly referred to as treatment-emergent neuroendocrine prostate cancer (t-NEPC) [4].

Treatment-emergent NEPC typically develops during the course of mCRPC after exposure to ADT and AR-targeted therapies and is characterized by rapid progression, visceral or atypical metastatic spread, reduced dependence on AR signaling, and poor survival [4,6,7]. Biopsy-based studies suggest that neuroendocrine transformation may be detected in approximately 10–20% of patients with progressive mCRPC, particularly after exposure to potent AR-targeted agents [4,7,8,9]. These findings support t-NEPC as a clinically relevant and frequently underrecognized mechanism of resistance in the modern treatment era [4,8,9].

Histopathologic examination of metastatic tumor tissue remains the reference standard for diagnosing t-NEPC and may reveal pure or mixed small-cell or large-cell neuroendocrine morphology, often accompanied by neuroendocrine marker expression [4,10]. However, metastatic biopsy is not always feasible in heavily pretreated patients with advanced disease, and intrapatient tumor heterogeneity may limit the diagnostic yield of a single tissue sample [6,7]. Moreover, differences in terminology and diagnostic thresholds across pathology, clinical oncology, and translational research have contributed to variability in the reported frequency and recognition of neuroendocrine transformation [11].

In this context, aggressive-variant prostate cancer (AVPC) provides a complementary clinical framework for recognizing rapidly progressive, AR-independent disease when histopathologic confirmation is unavailable [4]. It is a composite clinicopathologic category characterized by aggressive clinical features, relative resistance to AR-targeted therapies, and potential sensitivity to platinum-based chemotherapy [4,12]. Typical features include exclusive or predominant visceral metastases, predominantly lytic bone metastases, bulky disease, prostate-specific antigen (PSA) levels discordant with tumor burden, elevated lactate dehydrogenase (LDH) or carcinoembryonic antigen (CEA) levels, a short duration of response to ADT, and/or small-cell neuroendocrine histology [12]. Histologically confirmed t-NEPC and clinically defined AVPC are overlapping but nonidentical entities, and AVPC does not require neuroendocrine morphology [4]. When metastatic biopsy is not feasible, overlapping clinical, biochemical, and radiographic features may raise clinical suspicion for a clinically defined t-NEPC/AVPC-like phenotype; however, this should be regarded as a clinical suspicion category rather than a substitute for histopathologic diagnosis.

Therapeutic options for t-NEPC and AVPC-like disease remain limited. Platinum-based chemotherapy is commonly used in this setting, largely by extrapolation from small-cell lung cancer and studies of clinically defined AVPC [4]. Patients with histologically confirmed NEPC are frequently treated with platinum–etoposide or platinum–taxane regimens; however, responses are often transient and survival remains poor [4,7,13]. In a pooled analysis of NEPC progressing from conventional prostate adenocarcinoma, median survival after NEPC diagnosis was approximately 7 months [13]. More recent real-world data have also demonstrated heterogeneous and generally limited outcomes with carboplatin–etoposide, particularly in heavily pretreated patients without histologically confirmed small-cell disease [14]. Although prospective studies have enrolled and treated patients on the basis of clinical AVPC criteria without requiring histopathologic confirmation in every case, much of the available evidence has been derived from biopsy-confirmed NEPC cohorts or prospectively defined AVPC populations. In clinical practice, however, metastatic biopsy may be unavailable or impractical in heavily pretreated patients with rapidly progressive disease. Consequently, the clinical characteristics, treatment patterns, survival outcomes, and routinely available prognostic factors of patients managed as having a clinically defined t-NEPC/AVPC-like phenotype remain insufficiently characterized [7,12,15].

To address this real-world evidence gap, we aimed to characterize the clinical features, neuroendocrine-directed treatment patterns, and survival outcomes of patients who were clinically identified as having a t-NEPC/AVPC-like phenotype during mCRPC and to evaluate routinely available clinical and laboratory factors associated with overall survival. By focusing on a clinically defined population encountered in routine practice, this study complements previous biopsy-based t-NEPC series and prospective studies using predefined AVPC criteria.

## 2. Materials and Methods

### 2.1. Study Design and Patient Population

This retrospective, single-center study included patients with prostate cancer who were followed and treated at Hacettepe University Cancer Institute between 1 January 2010 and 31 December 2020. The institutional database contained records of 354 patients with prostate cancer for whom clinical, pathologic, treatment-related, and follow-up data were available.

Metastatic CRPC was defined according to Prostate Cancer Working Group 2 (PCWG2) recommendations as radiologically documented metastatic disease with progression despite ongoing ADT or surgical castration and castrate serum testosterone levels (<50 ng/dL), when available [16]. Metastatic disease was defined by radiographic evidence of distant metastases on conventional imaging.

Because metastatic biopsy was not routinely feasible in patients with advanced disease, patients considered to have a clinically defined t-NEPC/AVPC-like phenotype during the course of mCRPC were identified on the basis of a constellation of clinical, biochemical, radiographic, and, when available, histopathologic features described in previous studies [4,7,12,13]. These features included rapid clinical or radiographic progression, early development of castration resistance, visceral or unusual metastatic spread, bulky disease, low or discordant PSA levels relative to tumor burden, elevated LDH or CEA levels, elevated serum neuroendocrine markers when available, and histologic evidence of small-cell or neuroendocrine differentiation when metastatic biopsy had been performed. No formal minimum number of features or weighted scoring system was applied; classification reflected the integrated clinical assessment of the treating oncologist, as documented in the medical records.

Patients were eligible if they had an initial diagnosis of prostate adenocarcinoma, subsequently developed mCRPC, were clinically identified as having a t-NEPC/AVPC-like phenotype based on the above features, and had platinum- and/or etoposide-based neuroendocrine-directed systemic therapy planned. Patients with de novo small-cell or neuroendocrine prostate carcinoma at initial diagnosis and those with insufficient clinical or follow-up data were excluded.

For outcome analyses, the index date was defined as the date of neuroendocrine-directed treatment initiation. For patients who did not initiate the planned treatment because of rapid clinical deterioration or patient preference, the date of the documented treatment decision was used as the index date. Overall, 74 patients met the eligibility criteria and constituted the final study cohort.

### 2.2. Clinical Data Collection

Clinical, demographic, pathologic, laboratory, and treatment-related data were retrospectively collected from institutional electronic medical records and patient files. Variables related to the initial diagnosis of prostate cancer included age, Gleason score, PSA level, and the presence of de novo metastatic disease. Additional baseline variables included prior prostate-directed local treatments, history of surgical castration, and comorbidities. Prostate-directed local treatments included radical prostatectomy and definitive or salvage radiotherapy.

Clinical characteristics at the index date included age, metastatic distribution, laboratory parameters, and the number of prior systemic treatment lines. Metastatic involvement was categorized as bone, lymph node, visceral or other atypical metastatic involvement. The specific sites of metastatic involvement were also recorded.

Laboratory parameters obtained at or immediately before the index date included PSA, hemoglobin, albumin, LDH, CEA, and serum neuroendocrine markers, when available. Prior disease-directed treatments administered before the index date, including ADT, ARPIs, taxane-based chemotherapy, radiotherapy, and lutetium-177–prostate-specific membrane antigen (^177^Lu-PSMA) radioligand therapy, were also documented.

Time intervals were calculated using clinically documented dates. The interval from initial prostate cancer diagnosis to the index date was calculated from the date of histopathologic diagnosis of prostate cancer. The interval from metastatic disease to the index date was calculated from the first radiographic documentation of distant metastasis. The interval from CRPC to the index date was calculated from the date of documented castration-resistant progression.

### 2.3. Neuroendocrine-Directed Treatment Data and Classification 

Neuroendocrine-directed treatment approaches were recorded for patients with a clinically defined t-NEPC/AVPC-like phenotype. Treatment selection was determined at the discretion of the treating medical oncologist based on clinical fitness, disease burden, prior treatment exposure, organ function, and patient preference.

Neuroendocrine-directed systemic therapy included platinum–etoposide combination chemotherapy, oral etoposide monotherapy, platinum monotherapy, and other platinum-containing combination regimens. Patients for whom systemic therapy was planned but not initiated because of rapid clinical deterioration or patient preference were retained in the overall cohort but excluded from treatment-group comparisons.

For descriptive and exploratory comparative analyses, first-line neuroendocrine-directed treatment was categorized as doublet chemotherapy or monotherapy. Doublet chemotherapy included platinum–etoposide and other platinum-containing combination regimens, whereas monotherapy included oral etoposide and platinum monotherapy. The administered regimen, number of treatment cycles, line of neuroendocrine-directed therapy, and treatment initiation status were recorded for each patient.

### 2.4. Outcome Definitions and Assessments

Overall survival (OS) was defined as the time from the index date to death from any cause, with patients alive at the last documented follow-up censored on that date. Radiographic progression-free survival (rPFS) and PSA progression-free survival (PSA-PFS) were defined as the time from the index date to radiographic or PSA progression, respectively, or death from any cause, whichever occurred first. Patients without progression or death were censored at the date of the last available radiographic assessment or PSA measurement, as appropriate.

Soft-tissue response and progression in patients with measurable nodal and/or visceral disease were assessed according to the Response Evaluation Criteria in Solid Tumors version 1.1 (RECIST 1.1) [17]. Radiographic response analyses were restricted to patients with measurable disease at baseline and at least one post-index imaging assessment. Bone progression was assessed using available conventional imaging studies, including bone scintigraphy, computed tomography, and/or magnetic resonance imaging, together with contemporaneous institutional radiology reports. When serial bone scintigraphy was available, progression was interpreted according to PCWG2 principles [16]; in patients without serial bone scintigraphy, osseous progression was based on radiologist-documented new or unequivocally progressive osseous lesions on computed tomography or magnetic resonance imaging.

PSA progression was defined as a ≥25% increase from the nadir value, with an absolute increase of at least 2 ng/mL, confirmed by a second measurement obtained at least three weeks later [16]. A PSA response was defined as a decline of ≥50% from the baseline PSA level.

Because of the retrospective study design and variability in imaging and laboratory assessment schedules, radiographic response and PSA-based outcomes were evaluated only in patients with sufficient longitudinal data. PSA-based outcomes were considered exploratory because PSA kinetics may not reliably reflect disease burden in patients with a clinically defined t-NEPC/AVPC-like phenotype.

### 2.5. Statistical Analysis

Descriptive statistics were used to summarize patient characteristics and treatment patterns. Continuous variables were reported as medians with first and third quartiles (Q1–Q3) or minimum and maximum values, as appropriate, and categorical variables were presented as frequencies and percentages. Missing data were handled using available-case analysis, and no imputation was performed.

Survival outcomes were estimated using the Kaplan–Meier method and reported with 95% confidence intervals (CIs). Median follow-up duration was estimated using the reverse Kaplan–Meier method. Differences between groups were assessed using the log-rank test. Comparisons between doublet chemotherapy and monotherapy were exploratory and unadjusted. Patients who did not initiate neuroendocrine-directed systemic therapy were excluded from treatment-group comparisons.

Factors associated with overall survival were initially evaluated using univariable Cox proportional hazards regression analyses. Variables considered clinically relevant and/or associated with OS at *p* < 0.10 in univariable analyses were considered for inclusion in the multivariable Cox regression model. To reduce the risk of overfitting, the final number of covariates was limited on the basis of the sample size and number of observed death events. Hazard ratios (HRs) were reported with 95% CIs.

Categorical thresholds used in the Cox regression analyses were selected on the basis of established clinical classifications, previously published prognostic studies, or clinically interpretable thresholds. Gleason scores of 9–10 were grouped together because they correspond to ISUP Grade Group 5, representing the highest histologic grade category [2]. The hemoglobin threshold of <12 g/dL was selected based on its previous use as a prognostic cut-off in mCRPC [18], whereas albumin <3.5 g/dL was used as a commonly used clinical threshold for hypoalbuminemia and has also been used in prognostic stratification of patients with mCRPC [19]. The 36-month cut-off for the interval from metastatic disease to the index date was selected as a clinically interpretable 3-year threshold and was not intended to represent a previously validated prognostic cut-off.

All statistical tests were 2-sided, and *p* < 0.05 was considered statistically significant. Statistical analyses were performed using IBM SPSS Statistics for Windows, version 23.0 (IBM Corp., Armonk, NY, USA).

## 3. Results

### 3.1. Baseline Characteristics and Prior Treatment Exposure

**Figure 1:**
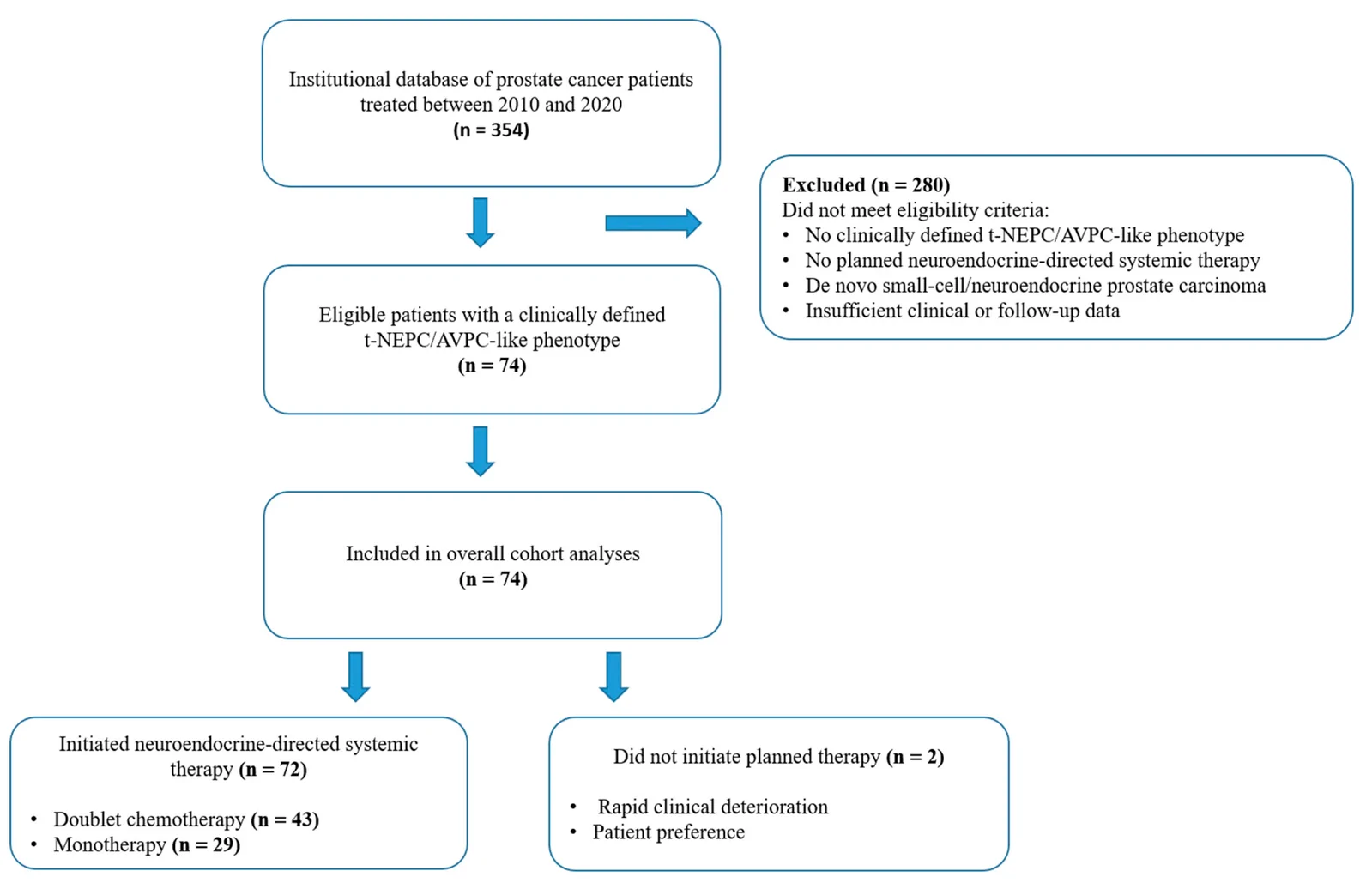
Patient flow diagram. Of 354 patients in the institutional prostate cancer database, 74 met the eligibility criteria for a clinically defined t-NEPC/AVPC-like phenotype and were included in the overall cohort analyses. Neuroendocrine-directed systemic therapy was initiated in 72 patients; 43 received doublet chemotherapy and 29 received monotherapy. Two patients did not initiate the planned treatment because of rapid clinical deterioration or patient preference.

Of the 354 patients screened in the institutional database, 74 (20.9%) met the study eligibility criteria and constituted the final clinically defined t-NEPC/AVPC-like cohort (Figure 1). The median age at initial prostate cancer diagnosis was 60.7 years (Q1–Q3, 54.3–67.6), and the median PSA level was 59.7 ng/mL (Q1–Q3, 15.5–179.0). De novo metastatic disease was present in 68.9% of patients, and 60.8% had a Gleason score ≥9. Prior prostate-directed local treatment included radical prostatectomy in 12.2% and definitive or salvage radiotherapy in 27.0%; 18.9% had undergone orchiectomy as a method of surgical castration.

Before the index date, patients had received multiple lines of systemic therapy. Docetaxel was the most frequently administered first-line treatment, used in 67 patients (90.5%). Among the 69 patients who received second-line therapy, cabazitaxel and abiraterone acetate were the most frequently used agents, administered to 27 (39.1%) and 23 (33.3%) patients, respectively. Cabazitaxel was the most common third-line treatment, administered to 13 of 38 patients (34.2%). Of the 17 patients who received fourth-line therapy, 10 (58.8%) received ^177^Lu-PSMA radioligand therapy, the most common treatment in this line. Baseline clinical characteristics and prior systemic treatment exposure are summarized in Table 1.

### 3.2. Clinical Characteristics at the Time of Clinical Identification of the t-NEPC/AVPC-like Phenotype

At the index date, the median age was 66.4 years (Q1–Q3, 60.3–72.3), and 47 patients (63.5%) were younger than 70 years. The median PSA level was 192.5 ng/mL (Q1–Q3, 53.9–714.5), and the median LDH level was 533 U/L (Q1–Q3, 369–1041). Among patients with available measurements, the median CEA and NSE levels were 11.1 ng/mL (Q1–Q3, 2.7–36.7) and 44 ng/mL (Q1–Q3, 24.0–62.7), respectively.

The median interval from initial prostate cancer diagnosis to the index date was 47.5 months (Q1–Q3, 28.4–99.3). The corresponding intervals from the first documentation of metastatic disease and from the development of CRPC to the index date were 38.1 months (Q1–Q3, 24.0–56.8) and 22.7 months (Q1–Q3, 12.0–37.9), respectively. Patients had received a median of three prior lines of systemic therapy (minimum–maximum, 1–5) before the index date.

Bone and lymph node metastases were present in 69 (93.2%) and 58 (78.4%) patients, respectively. Visceral metastases were documented in 50 patients (67.6%), most commonly involving the liver (*n* = 41, 55.4%) and lung (*n* = 16, 21.6%). Additional atypical metastatic sites included the central nervous system, pleura, orbit, peritoneum, adrenal glands, spleen, and bone marrow, with a total of 20 site-specific occurrences recorded. The median hemoglobin level was 10.5 g/dL (Q1–Q3, 9.4–11.6), and hypoalbuminemia, defined as a serum albumin level <3.5 g/dL, was present in 26 of 72 evaluable patients (36.1%). Clinical and laboratory characteristics at the index date are summarized in Table 2.

### 3.3. Neuroendocrine-Directed Systemic Therapy

Neuroendocrine-directed systemic therapy was planned for all 74 patients after clinical identification of the t-NEPC/AVPC-like phenotype. Treatment was initiated in 72 patients (97.3%), whereas two patients (2.7%) did not receive the planned therapy because of rapid clinical deterioration or patient preference. Among the 72 treated patients, 43 (59.7%) received doublet chemotherapy and 29 (40.3%) received monotherapy.

In the overall cohort (*n* = 74), platinum–etoposide was the most frequently administered first-line neuroendocrine-directed regimen, used in 41 patients (55.4%), followed by oral etoposide monotherapy in 21 patients (28.4%). Other platinum-based regimens, including carboplatin monotherapy and carboplatin–paclitaxel, were administered to 10 patients (13.5%). First-line neuroendocrine-directed treatment patterns are summarized in Table 3.

Twenty-nine patients subsequently received second-line neuroendocrine-directed systemic therapy. Irinotecan was the most frequently administered second-line treatment, used in 10 of 29 patients (34.5%). The remaining patients received heterogeneous regimens, including oral etoposide and additional platinum-based therapies.

### 3.4. Survival Outcomes

**Figure 2:**
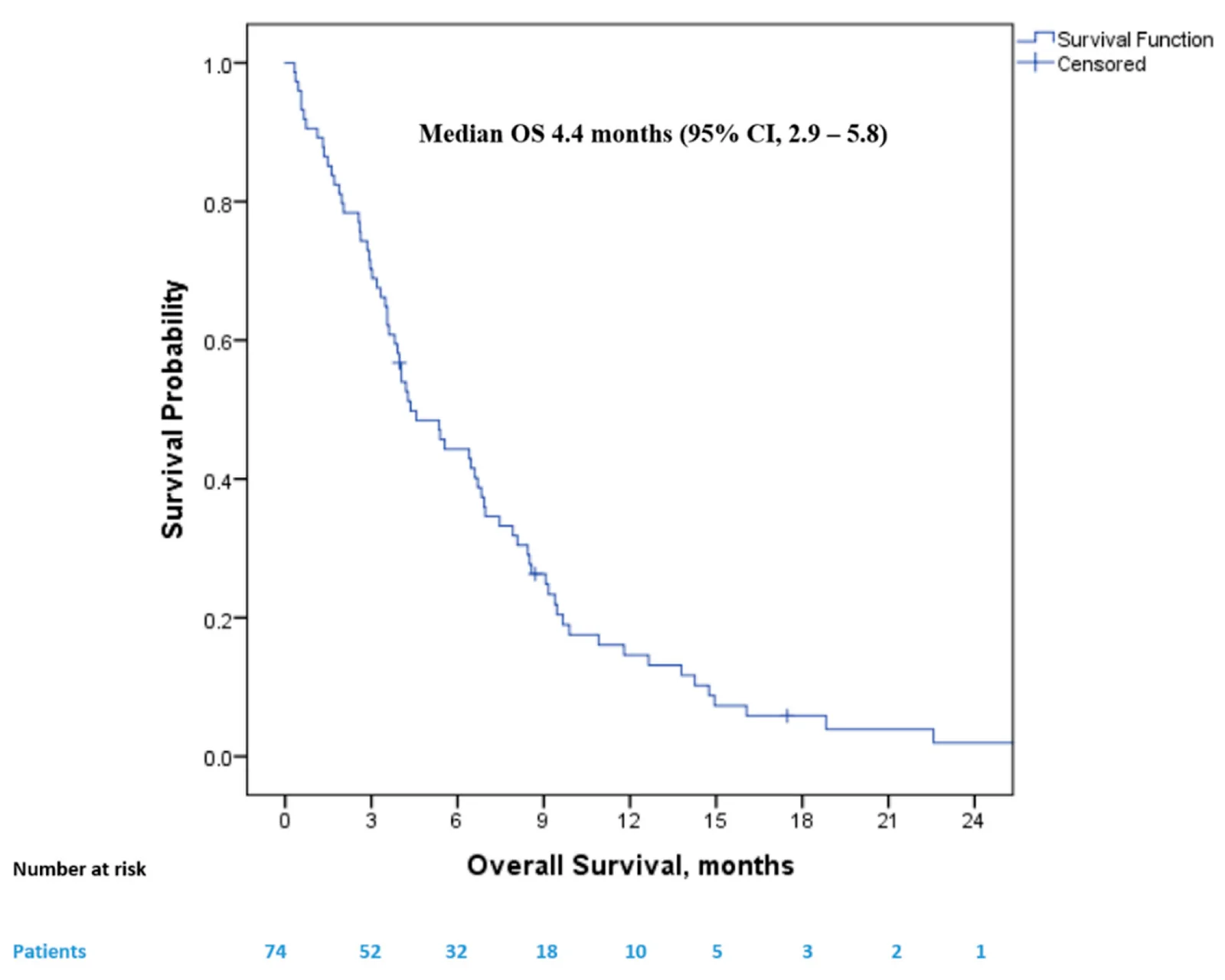
Kaplan–Meier estimate of overall survival from the clinical identification of the clinically defined t-NEPC/AVPC-like phenotype. Median overall survival was 4.4 months (95% CI, 2.9–5.8). Tick marks indicate censored observations. Numbers at risk are shown below the x-axis.

**Figure 3:**
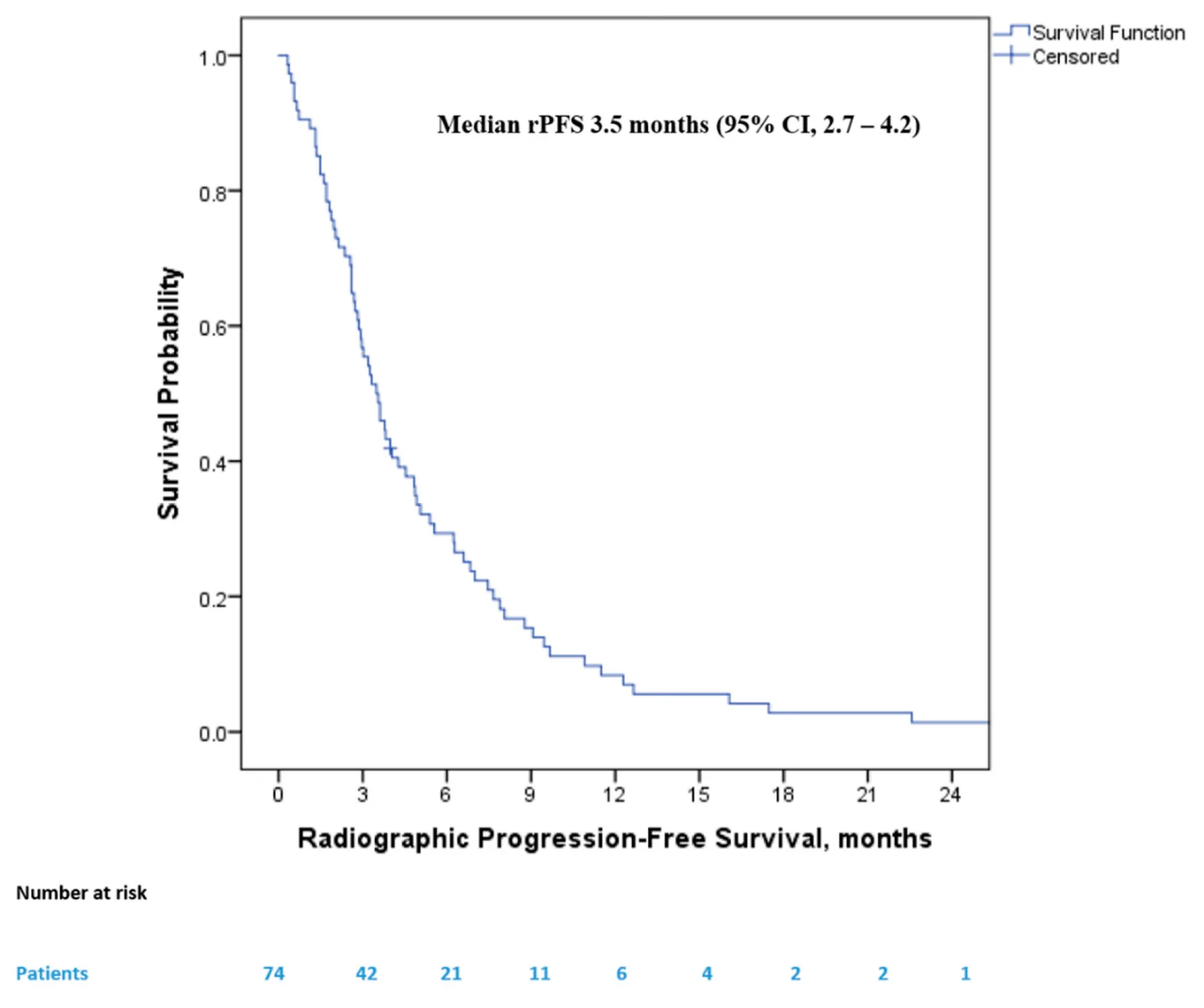
Kaplan–Meier estimate of radiographic progression-free survival from the clinical identification of the clinically defined t-NEPC/AVPC-like phenotype. Median radiographic progression-free survival was 3.5 months (95% CI, 2.7–4.2). Tick marks indicate censored observations. Numbers at risk are shown below the x-axis.

Patients were followed from the index date until death or last follow-up. The median follow-up duration, estimated using the reverse Kaplan–Meier method, was 4.3 months (Q1–Q3, 2.6–8.7). In the overall cohort, the median OS was 4.4 months (95% CI, 2.9–5.8) (Figure 2). The estimated 3-, 6-, and 12-month OS rates were 68.9%, 42.9%, and 14.6%, respectively. The median rPFS was 3.5 months (95% CI, 2.7–4.2) (Figure 3).

**Figure 4:**
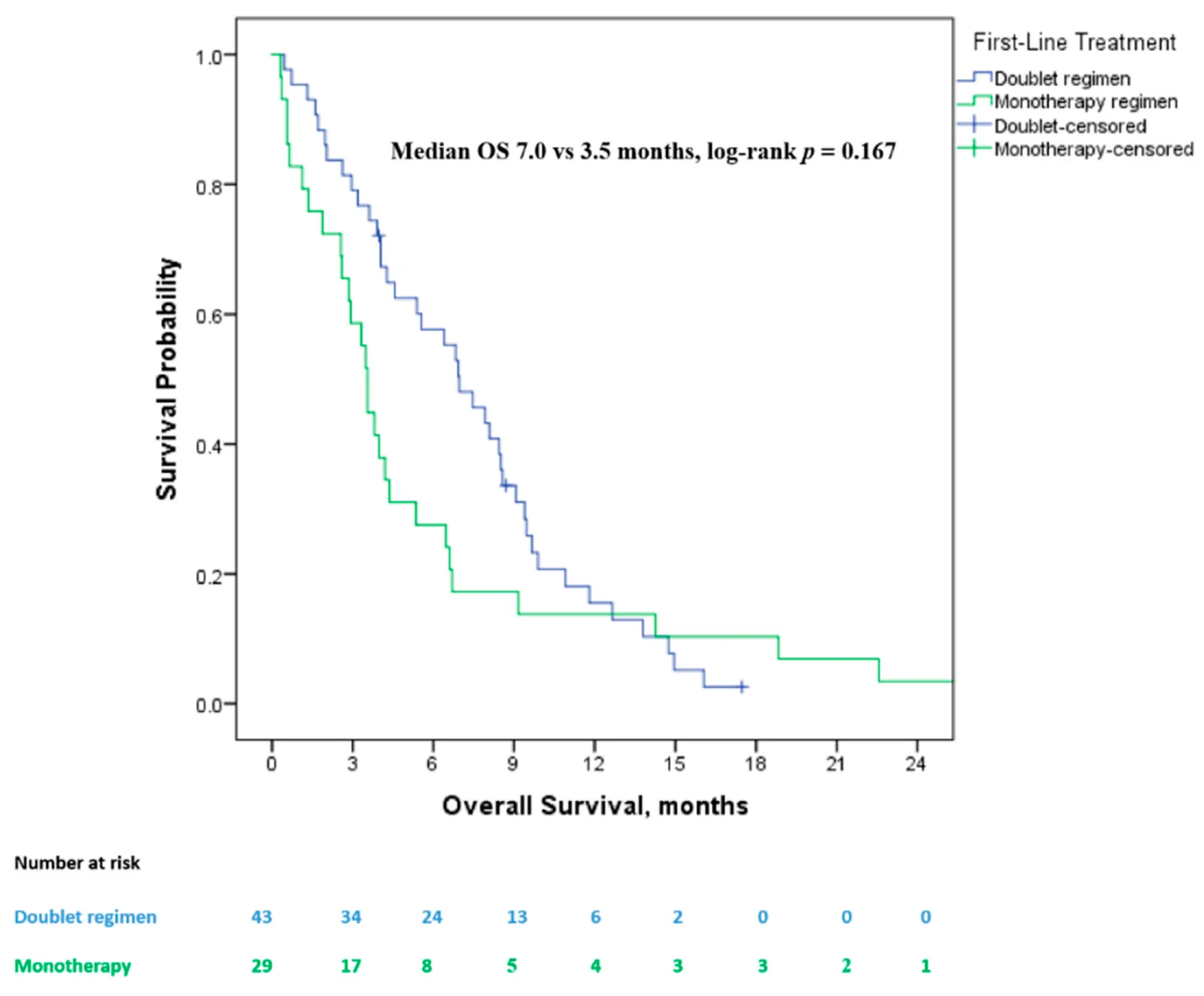
Kaplan–Meier estimates of overall survival from the initiation of first-line neuroendocrine-directed systemic therapy, stratified by treatment regimen. Median overall survival was 7.0 months in the doublet chemotherapy group and 3.5 months in the monotherapy group (log-rank *p* = 0.167). Tick marks indicate censored observations. Numbers at risk are shown below the x-axis.

**Figure 5:**
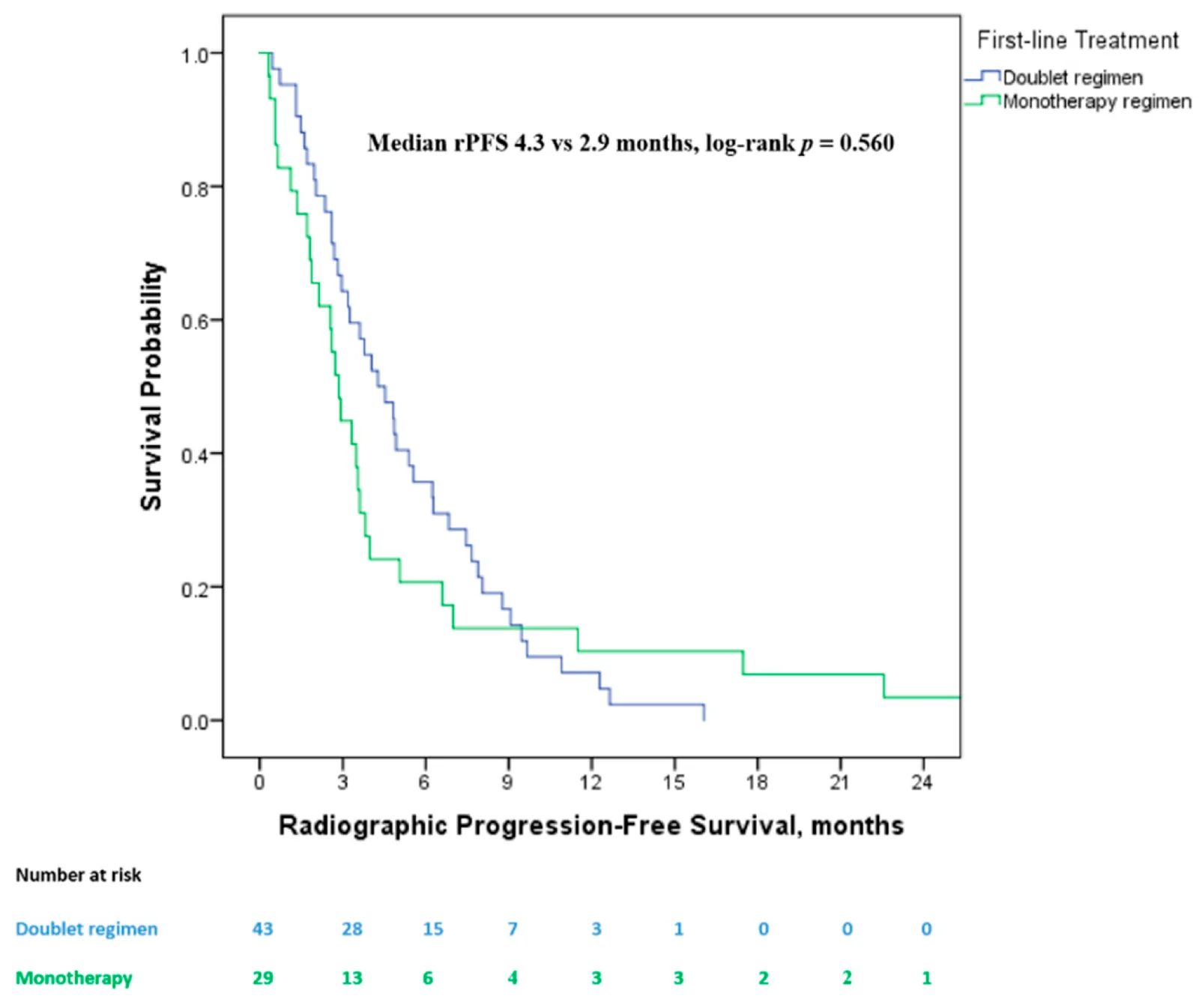
Kaplan–Meier estimates of radiographic progression-free survival from the initiation of first-line neuroendocrine-directed systemic therapy, stratified by treatment regimen. Median radiographic progression-free survival was 4.3 months (95% CI, 3.0–5.6) in the doublet chemotherapy group and 2.9 months (95% CI, 2.3–3.4) in the monotherapy group (log-rank *p* = 0.560). Numbers at risk are shown below the x-axis.

In an exploratory analysis restricted to the 72 patients who initiated neuroendocrine-directed systemic therapy, median OS was numerically longer with doublet chemotherapy than with monotherapy (7.0 vs. 3.5 months; log-rank *p* = 0.167) (Figure 4). Median rPFS was 4.3 months (95% CI, 3.0–5.6) in the doublet chemotherapy group and 2.9 months (95% CI, 2.3–3.4) in the monotherapy group, with no statistically significant difference between groups (log-rank *p* = 0.560) (Figure 5).

PSA-based outcomes were considered exploratory. The median PSA-PFS was 2.6 months (95% CI, 2.1–3.1). Among 71 patients with evaluable serial PSA measurements, 10 (14.1%) achieved a PSA response, 28 (39.4%) had stable PSA, and 33 (46.5%) experienced PSA progression during first-line neuroendocrine-directed therapy.

### 3.5. Prognostic Factors Associated with Overall Survival

In univariable Cox regression analyses, Gleason score ≥9 (HR, 1.88; 95% CI, 1.03–3.43; *p* = 0.040), absence of prior orchiectomy (HR, 2.22; 95% CI, 1.15–4.27; *p* = 0.018), hemoglobin level <12 g/dL (HR, 2.22; 95% CI, 1.17–4.20; *p* = 0.015), albumin level <3.5 g/dL (HR, 2.88; 95% CI, 1.67–4.98; *p* < 0.001), liver metastasis (HR, 1.94; 95% CI, 1.18–3.20; *p* = 0.010), and central nervous system metastasis (HR, 3.63; 95% CI, 1.41–9.32; *p* = 0.007) were associated with worse OS. An interval of <36 months from the first documentation of metastatic disease to the index date was also associated with an increased risk of death (HR, 2.23; 95% CI, 1.31–3.81; *p* = 0.003). Age at initial prostate cancer diagnosis and the first-line neuroendocrine-directed treatment approach (doublet chemotherapy vs. monotherapy) were not significantly associated with OS.

To reduce the risk of overfitting, the multivariable model was restricted to five clinically relevant variables: Gleason score, hemoglobin level, albumin level, liver metastasis, and the interval from metastatic disease to index date. After adjustment, Gleason score ≥9 (HR, 2.13; 95% CI, 1.03–4.40; *p* = 0.041), hemoglobin level <12 g/dL (HR, 2.22; 95% CI, 1.01–4.87; *p* = 0.047), and albumin level <3.5 g/dL (HR, 2.15; 95% CI, 1.16–3.96; *p* = 0.015) remained independently associated with worse OS. Liver metastasis (HR, 1.54; 95% CI, 0.87–2.72; *p* = 0.140) and a metastatic disease–index date interval of <36 months (HR, 1.26; 95% CI, 0.66–2.43; *p* = 0.484) were not independently associated with OS. Central nervous system metastasis was not included in the multivariable model because of the small number of affected patients (*n* = 5). The complete Cox regression results are presented in Table 4.

## 4. Discussion

In this single-center retrospective study, we evaluated the clinical characteristics, treatment patterns, and outcomes of patients with a clinically defined t-NEPC/AVPC-like phenotype arising during mCRPC. The cohort had a substantial metastatic burden and poor outcomes despite neuroendocrine-directed systemic therapy. From the time of clinical identification of the phenotype, median OS and rPFS were 4.4 and 3.5 months, respectively. In the exploratory, unadjusted analysis, doublet chemotherapy was associated with numerically longer OS than monotherapy, although the difference was not statistically significant. These findings underscore the aggressive clinical course of this phenotype and the need for earlier recognition and more effective therapeutic strategies.

Treatment-emergent neuroendocrine prostate cancer is increasingly recognized as an important mechanism of resistance in mCRPC [6]. Although biopsy-based studies have reported neuroendocrine or small-cell transformation in approximately 10–20% of patients with mCRPC, the frequency recognized in routine practice is likely lower because metastatic biopsies are not routinely performed and clinical recognition remains inconsistent [4,5,20]. Our cohort represents a real-world population in whom a t-NEPC/AVPC-like phenotype was identified using a constellation of clinical, biochemical, and radiographic features. Although metastatic tissue biopsy remains the diagnostic reference standard, it is often not feasible in patients with advanced disease, and no noninvasive biomarker has been established for routine clinical use [4,5,10,21]. Accordingly, this clinically defined approach reflects pragmatic decision-making in heavily pretreated patients but should be regarded as a clinical suspicion framework rather than a substitute for histopathologic diagnosis.

The clinical presentation of our cohort was broadly consistent with previous biopsy-confirmed NEPC and AVPC series [7,12]. Visceral metastases were present in approximately two-thirds of patients, with the liver being the most frequently involved visceral site. Uncommon metastatic sites, including the central nervous system, pleura, and peritoneum, were also observed. Notably, the median PSA level at clinical identification was substantially elevated, contrasting with the classical description of NEPC as a low-PSA disease. This finding likely reflects the broader and biologically heterogeneous spectrum captured by a clinically defined t-NEPC/AVPC-like phenotype, in which PSA may be discordant with disease burden but is not necessarily low. In patients with progressive mCRPC, suspicion for a t-NEPC/AVPC-like phenotype should be heightened when rapid or discordant progression occurs, particularly in the presence of visceral, especially hepatic, or atypical metastases, bulky or predominantly lytic disease, PSA kinetics disproportionate to radiographic tumor burden, or elevations in LDH, CEA, or neuroendocrine markers [4,7,12]. When these features raise clinical suspicion, metastatic biopsy should be considered whenever clinically feasible.

Another clinically relevant observation was the disease trajectory preceding clinical identification of the t-NEPC/AVPC-like phenotype [7,13]. In the cohort reported by Conteduca et al., 30.0% of patients with therapy-related NEPC presented with de novo metastatic disease, and the median interval from prostate adenocarcinoma diagnosis to NEPC diagnosis was 39.7 months [7]. Early emergence may also occur, as illustrated by a recent case report of NEPC developing shortly after triplet therapy despite marked PSA suppression, with focal neuroendocrine differentiation in the initial biopsy suggesting a pre-existing neuroendocrine component [22]. In our cohort, de novo metastatic disease was more frequent, occurring in 68.9% of patients. The median intervals from the first documentation of metastatic disease and from CRPC development to clinical identification of the t-NEPC/AVPC-like phenotype were 38.1 and 22.7 months, respectively. Thus, despite the high proportion of patients presenting with metastatic disease, the clinically defined phenotype generally became apparent only after a prolonged metastatic and heavily treated disease course. Cross-study comparisons should nevertheless be made cautiously because clinical identification in our study reflected the treatment initiation or decision time point rather than histopathologic confirmation or the biologic onset of lineage transformation.

The prognosis of t-NEPC is generally poor, although reported survival varies according to histologic composition and disease extent, and study population [4]. In the pooled analysis by Wang et al., median OS after NEPC diagnosis was approximately 7 months, and a greater number of metastatic organs was independently associated with worse survival [13]. Zhang et al. reported a median OS of 17.6 months in their overall pathologically confirmed t-NEPC cohort, whereas Aparicio et al. reported a median OS of 16 months in patients with clinically defined AVPC treated with platinum-based chemotherapy [12,23]. In our cohort, median OS was shorter, at 4.4 months. This difference may reflect the advanced and heavily pretreated population, including the high prevalence of visceral and liver metastases, a median of three prior systemic treatment lines, and differences in baseline fitness and treatment selection.

No standard systemic therapy has been established for t-NEPC, although platinum-based chemotherapy is commonly used in patients considered fit for treatment [4,5,14]. Platinum–etoposide and platinum–taxane regimens have shown activity in heterogeneous populations with AVPC, biopsy-confirmed NEPC, or CRPC with neuroendocrine features [4,12]. Aparicio et al. demonstrated activity with platinum-based chemotherapy in patients meeting clinical AVPC criteria, whereas Baude et al. reported median PFS and OS of 3.7 and 7.7 months, respectively, with carboplatin–etoposide in heavily pretreated adenocarcinoma with elevated neuroendocrine markers [12,14]. In our cohort, median OS and rPFS were numerically longer with doublet chemotherapy than with monotherapy (7.0 vs. 3.5 months and 4.3 vs. 2.9 months, respectively), but neither difference was statistically significant. Because treatment selection was nonrandomized, these exploratory, unadjusted comparisons should not be interpreted as evidence of treatment superiority. The median PSA-PFS of 2.6 months further illustrates the limited durability of current treatment strategies.

Beyond the overall poor prognosis of this phenotype, multivariable analysis identified Gleason score ≥9, hemoglobin <12 g/dL, and albumin <3.5 g/dL as independent adverse prognostic factors for OS. High Gleason score likely reflects aggressive tumor biology [7,13], whereas anemia and hypoalbuminemia may indicate advanced disease, systemic inflammation, nutritional impairment, and reduced physiologic reserve [24]. These routinely available parameters may support clinical risk stratification, but their prognostic value requires validation in independent cohorts.

Given the diagnostic challenges associated with clinically defined t-NEPC/AVPC-like disease, noninvasive strategies that may support earlier recognition are urgently needed [21]. Circulating tumor cell-based transcriptomic assays and extracellular vesicle-derived molecular signatures may help identify neuroendocrine differentiation when metastatic tissue sampling is not feasible, although these approaches remain investigational [21,25]. Concurrent loss of RB1 and TP53, together with activation of drivers such as AURKA and MYCN, has been implicated in neuroendocrine differentiation and androgen receptor-independent disease evolution [4,26]. These findings may inform future biomarker-driven diagnostic and therapeutic strategies but require prospective clinical validation [27,28].

Several limitations should be acknowledged. First, the retrospective, single-center design introduces the potential for selection bias, incomplete data capture, and limited generalizability. Second, metastatic biopsy confirmation was not routinely available, and classification based on clinical, biochemical, and radiographic features may have resulted in diagnostic heterogeneity or misclassification. Third, treatment selection was nonrandomized, making the comparison between doublet chemotherapy and monotherapy susceptible to confounding by indication. Imaging and laboratory assessments were also not performed at standardized intervals, which may have affected rPFS, PSA-PFS, and response evaluations. Finally, the relatively small sample size limited statistical power, constrained the multivariable model, and precluded detailed subgroup analyses. Despite these limitations, this study provides real-world data on an underrecognized and highly aggressive phenotype encountered when metastatic tissue confirmation is not feasible.

## 5. Conclusions

The clinically defined t-NEPC/AVPC-like phenotype represents a highly aggressive form of resistance arising during the course of mCRPC. In this real-world cohort, survival after clinical identification was poor despite neuroendocrine-directed systemic therapy. Although doublet chemotherapy was associated with numerically longer survival than monotherapy, the exploratory comparison did not demonstrate treatment superiority. Gleason score ≥9, anemia, and hypoalbuminemia were independently associated with inferior OS. These findings highlight the need for earlier recognition, improved biomarker-based diagnostic strategies, and more effective therapies for this clinically challenging population.

## Figures and Tables

**Table 1:** Baseline Clinical Characteristics and Prior Treatment Exposure.

Characteristic	Overall Cohort (*n* = 74)
Age at initial prostate cancer diagnosis, years (median, Q1–Q3)	60.7 (54.3–67.6)
Age group <70 years ≥70 years	61 (82.4%) 13 (17.6%)
PSA level, ng/mL (median, Q1–Q3) *	59.7 (15.5–179.0)
De novo metastatic disease Yes No	51 (68.9%) 23 (31.1%)
Gleason score <9 ≥9 Missing	18 (24.3%) 45 (60.8%) 11 (14.9%)
Radical prostatectomy Yes No	9 (12.2%) 65 (87.8%)
Definitive or salvage radiotherapy Yes No	20 (27.0%) 54 (73.0%)
Orchiectomy Yes No	14 (18.9%) 60 (81.1%)
Number of comorbid conditions Absent 1 2 3 or more	25 (33.8%) 22 (29.7%) 17 (23.0%) 10 (13.5%)
Prior systemic therapies before the index date † 1st line (*n* = 74) Docetaxel Enzalutamide Abiraterone acetate 2nd line (*n* = 69) Cabazitaxel Abiraterone acetate Enzalutamide 3rd line (*n* = 38) Cabazitaxel Abiraterone acetate Docetaxel rechallenge 4th line (*n* = 17) ^177^Lu-PSMA radioligand therapy Enzalutamide Cabazitaxel	67 (90.5%) 4 (5.4%) 2 (2.7%) 27 (39.1%) 23 (33.3%) 10 (14.5%) 13 (34.2%) 9 (23.7%) 6 (15.8%) 10 (58.8%) 3 (17.6%) 2 (11.8%)

Data are presented as n (%) unless otherwise indicated. * PSA level at initial prostate cancer diagnosis was available for 59 patients. † Only the three most frequently administered treatments in each treatment line are shown. Percentages for each treatment line were calculated using the number of patients who received treatment in the corresponding line as the denominator. Percentages for all other categorical variables were calculated using the overall cohort as the denominator. PSA, prostate-specific antigen; Q1–Q3, first and third quartiles; ^177^Lu-PSMA, lutetium-177–prostate-specific membrane antigen.

**Table 2:** Clinical and Laboratory Characteristics at the Time of Clinical Identification of the t-NEPC/AVPC-like Phenotype.

Characteristic	Overall Cohort (*n* = 74)
Age at the index date, years (median, Q1–Q3)	66.4 (60.3–72.3)
Age group at the index date <70 years ≥70 years	47 (63.5%) 27 (36.5%)
PSA level, ng/mL (median, Q1–Q3)	192.5 (53.9–714.5)
CEA level, ng/mL (median, Q1–Q3) *	11.1 (2.7–36.7)
LDH level, U/L (median, Q1–Q3)	533 (369–1041)
NSE level, ng/mL (median, Q1–Q3) †	44 (24.0–62.7)
Hemoglobin level, g/dL (median, Q1–Q3)	10.5 (9.4–11.6)
Albumin level, g/dL (*n* = 72) <3.5 g/dL ≥3.5 g/dL	26 (36.1%) 46 (63.9%)
Prostate cancer diagnosis–index date interval, months (median, Q1–Q3)	47.5 (28.4–99.3)
Metastatic disease–index date interval, months (median, Q1–Q3)	38.1 (24.0–56.8)
CRPC–index date interval, months (median, Q1–Q3)	22.7 (12.0–37.9)
Prior systemic treatment lines, median (min–max)	3 (1–5)
Metastatic distribution Bone Yes No Lymph nodes Yes No Any visceral metastasis Yes No Lung Yes No Liver Yes No	69 (93.2%) 5 (6.8%) 58 (78.4%) 16 (21.6%) 50 (67.6%) 24 (32.4%) 16 (21.6%) 58 (78.4%) 41 (55.4%) 33 (44.6%)

Data are presented as median (Q1–Q3) or n (%), unless otherwise indicated. * CEA data were available for 34 patients. † NSE data were available for 20 patients. AVPC, aggressive variant prostate cancer; CEA, carcinoembryonic antigen; CRPC, castration-resistant prostate cancer; LDH, lactate dehydrogenase; min–max, minimum–maximum; NSE, neuron-specific enolase; PSA, prostate-specific antigen; Q1–Q3, first and third quartiles; t-NEPC, treatment-emergent neuroendocrine prostate cancer.

**Table 3:** First-Line Neuroendocrine-Directed Treatment Patterns.

Regimen	Overall Cohort (*n* = 74)
Platinum–etoposide	41 (55.4%)
Oral etoposide monotherapy	21 (28.4%)
Other platinum-based regimens *	10 (13.5%)
Treatment not initiated	2 (2.7%)

Data are presented as n (%), with percentages calculated using the overall cohort as the denominator. * Other platinum-based regimens included carboplatin monotherapy and carboplatin–paclitaxel.

**Table 4:** Univariable and Multivariable Cox Regression Analyses of Overall Survival.

Parameter	Univariable Analysis		Multivariable Analysis	
	HR (95% CI)	*p*-Value	HR (95% CI)	*p*-Value
Age at diagnosis (<70 vs. ≥70)	1.68 (0.91–3.11)	0.099		
Gleason score (<9 vs. ≥9)	1.88 (1.03–3.43)	0.040	2.13 (1.03–4.40)	0.041
Orchiectomy status (yes vs. no)	2.22 (1.15–4.27)	0.018		
Hemoglobin level (≥12 vs. <12)	2.22 (1.17–4.20)	0.015	2.22 (1.01–4.87)	0.047
Albumin level (≥3.5 vs. <3.5)	2.88 (1.67–4.98)	<0.001	2.15 (1.16–3.96)	0.015
Liver metastasis (no vs. yes)	1.94 (1.18–3.20)	0.010	1.54 (0.87–2.72)	0.140
CNS metastasis (no vs. yes)	3.63 (1.41–9.32)	0.007		
Metastatic disease–index date interval (≥36 vs. <36 months)	2.23 (1.31–3.81)	0.003	1.26 (0.66–2.43)	0.484
Treatment (doublet vs. monotherapy)	1.42 (0.86–2.35)	0.170		

The multivariable Cox regression model was restricted to five clinically relevant variables (Gleason score, hemoglobin level, albumin level, liver metastasis, and metastatic disease–index date interval) because of the limited number of observed death events. For each comparison, the first listed category was used as the reference; HR >1 indicates a higher risk of death for the second category relative to the first. CI, confidence interval; CNS, central nervous system; HR, hazard ratio.

## Data Availability

The datasets used and/or analysed during the current study are available from the corresponding author upon reasonable request.

## Abbreviations

ADT, androgen deprivation therapy; AR, androgen receptor; ARPI, androgen receptor pathway inhibitor; AVPC, aggressive variant prostate cancer; CEA, carcinoembryonic antigen; CI, confidence interval; CNS, central nervous system; CRPC, castration-resistant prostate cancer; HR, hazard ratio; LDH, lactate dehydrogenase; mCRPC, metastatic castration-resistant prostate cancer; NEPC, neuroendocrine prostate cancer; NSE, neuron-specific enolase; OS, overall survival; PCWG2, Prostate Cancer Working Group 2; PFS, progression-free survival; PSA, prostate-specific antigen; PSA-PFS, prostate-specific antigen progression-free survival; Q1–Q3, first and third quartiles; RECIST, Response Evaluation Criteria in Solid Tumors; rPFS, radiographic progression-free survival; t-NEPC, treatment-emergent neuroendocrine prostate cancer; ^177^Lu-PSMA, lutetium-177–prostate-specific membrane antigen.
